# Waiting to see the specialist: patient and provider characteristics of wait times from primary to specialty care

**DOI:** 10.1186/1471-2296-15-16

**Published:** 2014-01-25

**Authors:** Liisa Jaakkimainen, Richard Glazier, Jan Barnsley, Erin Salkeld, Hong Lu, Karen Tu

**Affiliations:** 1 Institute for Clinical Evaluative Sciences, 2075 Bayview Ave, G wing, Toronto, Ontario M4N 3M5, Canada; 2 Institute of Health Policy, Management and Evaluation, University of Toronto, 155 College Street, Suite 425, Toronto, Ontario M5T 3M6, Canada; 3 Central Local Health Integration Network, 60 Renfrew Drive, Suite 300, Markham, Ontario L3R 0E1, Canada

## Abstract

**Background:**

Wait times are an important measure of access to various health care sectors and from a patient’s perspective include several stages in their care. While mechanisms to improve wait times from specialty care have been developed across Canada, little is known about wait times from primary to specialty care. Our objectives were to calculate the wait times from when a referral is made by a family physician (FP) to when a patient sees a specialist physician and examine patient and provider factors related to these wait times.

**Methods:**

Our study used the Electronic Medical Record Administrative data Linked Database (EMRALD) which is a linkage of FP electronic medical record (EMR) data to the Ontario, Canada administrative data. The EMR referral date was linked to the administrative physician claims date to calculate the wait times. Patient age, sex, socioeconomic status, comorbidity and FP continuity of care and physician age, sex, practice location, practice size and participation in a primary care delivery model were examined with respect to wait times.

**Results:**

The median waits from medical specialists ranged from 39 to 76 days and for surgical specialists from 33 days to 66 days. With a few exceptions, patient factors were not associated with wait times from primary care to specialty care. Similarly physician factors were not consistently associated with wait times, except for FP practice location and size.

**Conclusions:**

Actual wait times for a referral from a FP to seeing a specialist physician are longer than those reported by physician surveys. Wait times from primary to specialty care need to be included in the calculation of surgical and diagnostic wait time benchmarks in Canada.

## Background

Wait times in Canada have focused on the time from seeing a specialist physician to having either an investigation or procedure, with the goal of improving access for a select number of health services such as cataract surgeries, cancer surgeries, cardiac procedures, hip and knee replacements and CT and MRI testing [1-4]. However, the wait times from a patient’s perspective included steps in care before they see a specialist physician or undergo an advanced diagnostic test. In fact, patients may face the greatest wait-related risk at the earlier stages of care before the disease has been fully characterized [5]. A patient’s pathway of care includes access to primary care, the wait time from a family physician (FP) referral to a visit with a specialist or the receipt of an investigation, the wait time from seeing the specialist to having a surgical procedure and the wait time where information from the specialist visit or surgical procedure is received back to the FP [6,7].

Patient socioeconomic status has been associated with less access to specialist care [8-14] and lower wait times [15]. Women and older patients are less likely to be referred for some specialist care [16-18]. Referral patterns for specialty care have been associated with practice locations and types of physician payment models [19-23]. Patient comorbidity has been associated with wait times for surgical procedure [24], but it has not been well examined for specialist referrals. There is little information about what patient or provider factors are associated with primary care wait times.

A Commonwealth survey of primary care physicians practicing in eleven countries found three-quarters of Canadian FPs reported long waits for specialist consultation and procedures [25]. Canada ranked 7th out of seven industrialized countries on timeliness of care, which included measures of wait times to and from primary care [26,27]. Currently in Canada, the only information on wait times from FP to specialists comes from patient or provider surveys [28-32]. However, data from these surveys are not objective measures and they are subject to response (with often less than 30% response rates) and recall bias.

Increasingly in Canada, electronic medical records (EMRs) are being used by FPs in their clinical practices, with enough uptake of EMR use to begin the process of developing methods for primary care research including the measurement of wait times [33-36]. However, extracting complete and accurate referral information from existing EMRs is challenging and similar work at a provincial or federal level is lacking [37-39].

Across Canada, administrative data have been used extensively to determine population level primary care performance [40-42]. It can describe care across health care sectors from physician offices to emergency room and inpatient care to community health care resources. However, administrative data are limited in its detail on what happens with a clinical encounter. EMR data do contain more detailed clinical information, but may not capture all encounters across health care sectors [34,39]. The linking of FP EMR data with administrative data provides the detail needed to describe the overall picture of the referral pathway between primary and specialty-based health care.

The objectives for this study were: 1) to calculate the wait times from when a referral is made by a FP to when a patient sees a medical or surgical consultant, and 2) to examine patient and provider factors related to these wait times.

## Methods

### Study design

Observational study of family medicine EMR data linked to health administrative data.

### Sources of data

The information used in this study came from the Electronic Medical Record Administrative data Linked Database (EMRALD) [38]. This database includes a linkage of FP EMR data using Practice Solutions® EMR to the Ontario administrative data held at the Institute for Clinical Evaluative Sciences (ICES). Practice Solutions® EMR is used by community-based Ontario FPs and it is the leading EMR software vendor across Ontario [43].

Medical and surgical specialist visits were identified using the Ontario Health Insurance Plan (OHIP) physician claims database held at ICES. At ICES methods to confidentially link individual level data across the multiple administrative data holdings were used.

### Study cohort

All patients who were alive as of December 31, 2008, had valid health care numbers, were rostered to a study FP, had a valid birth date and had at least one visit to their FP between January 1, 2008 and December 31, 2009 were included. With the introduction of Primary Care Reform in Ontario in 2003, patients became formally rostered to their FP who would be the main provider of their primary care [44]. This study included physicians who participated in EMRALD as of the January 2009 extraction and these physicians are distributed throughout Ontario.

### Wait times

Referral data for all study eligible patients was extracted from the EMR portion of the EMRALD database between January 1, 2008 and December 31, 2008. Referral data included a referral letter and date of the referral letter. The type of specialist referred to was not automatically coded. Therefore, a coding manual and data dictionary were developed for categorizing the content of the referral letter into referral specialist types. A file containing the referral date, referral specialist type, scrambled health care number and scrambled physician number was then uploaded and linked to the OHIP claims file. OHIP specialist claims for a full consultation were then identified. Follow up and reassessment visits were not included as they do not require a referral from a FP. The wait time was calculated from the date of the EMR referral to the date of the first OHIP consultation visit to the same or similar specialist type.

### Patient factors

Patient age, sex socioeconomic status, comorbidity and continuity of care with a family physician were examined with respect to wait times. Patient age and sex were determined from the Registered Persons Database (RPDB) held at ICES.

### Socioeconomic Status (SES)

A proxy measure for socioeconomic status was based on the ranking of each neighbourhood’s average household income compared to all other neighbourhoods in a given municipality [45]. These neighbourhood income quintiles were developed by Statistics Canada and have been used in multiple health administrative studies in Canada.

### Ambulatory Care Groups (ACGs)

For this study, the Johns Hopkins Adjusted Clinical Group (ACG) case-mix system was used as a measure of patient acuity/comorbidity [46]. The Johns Hopkins ACG system developed and validated a methodology based on the hypothesis that the clustering of morbidity is a better predicator of health services resource use than using the presence or absence of specific diseases alone [47]. The Johns Hopkins ACG system is based on patients’ diagnoses from physician visits and hospital admissions, which are assigned to one of 32 diagnosis clusters known as Aggregated Diagnosis Groups (ADGs). The number of ADGs a person had was summed and then grouped into acuity levels. Those with the greatest number of ADGs (in this case 10 or more) are the sickest and require the most healthcare resources.

### Usual Provider Continuity (UPC index)

Relational continuity examines sustained contact between a patient/client and a provider over time, with the UPC index being one measure [48]. High continuity of care with a FP has been associated with improved health outcomes, such as reduced ER use and hospital admissions [49]. The UPC index was calculated as the number of visits to the study FP over total number of visits to all FPs the patient had seen over a two year time frame. The UPC index was not calculated for patients having fewer than two visits. Patient were then categorized as having high continuity (UPC > =0.8), low continuity (UPC index < 0.8) or no continuity.

### Physician factors

Physician factors examined included physician age and sex, practice location and enrolment in a primary care delivery model. Physician age and sex were determined from the Corporate Provider Database (CPDB) held at ICES.

### Practice location

Practice location was defined using the Ontario Medical Association’s Rurality Index of Ontario (RIO) [50]. The RIO is based on community characteristics including travel time to different levels of care; community population; presence of providers, hospitals and ambulance services; social indicators; and weather conditions. The RIO was used to divide communities into major urban areas, non-major urban areas and rural areas.

Canada started to reform its primary care delivery system after the release of the Romanow report in 2002 [44]. In Ontario, the largest province in Canada, the Ministry of Health and Long-Term Care (MOHLTC) introduced new primary care enrolment models. In addition to the existing fee-for-service (FFS) model, there now are Family Health Groups (FHGs) which are a blended fee-for-service model, Family Health Networks (FHNs) which are a blended capitation model and Family Health Organizations (FHOs) which are entirely a capitation model. Capitation models include more formal rostering of patients to their FPs. Capitation models include different financial incentives for physicians (such as incentives for preventive care and chronic disease management) and they also include additional funding for interdisciplinary care. All residents of Ontario, Canada are eligible to enroll in any of these models. Even if they are enrolled in a model, patients are still able to see other FPs.

Physician group affiliations (primary care delivery models) were identified in the Client Agency Program Enrolment (CAPE) database of patient enrolments with primary care groups and the OHIP CPDB. The FPs were categorized as belonging to either a capitation-based model such as a FHO or FHN versus a mainly fee-for-service model such as FHG. We also examined the roster size for each study physician.

### Analysis

Wait times do not have a normal distribution. Therefore the descriptive analysis included the calculation of median and 75th percentiles [51]. Our study analyses were meant to be hypothesis generating. Bivariate analyses were undertaken to examine wait times in relation to patient and provider measures. Statistical testing was done using the Wilcoxon Rank Sum Test, median test and the Kruskal-Wallis One-Way AOV [52]. Only p values < 0.001 were considered statistically significant to correct for multiple testing. To examine whether any of these patient or providers had an independent association with wait times, multivariate linear regression using proc glm in SAS was done with a log transformation of wait times in days as the dependent variable and patient or provider characteristics as the independent variables [52,53].

This study had ethics approval from the Sunnybrook Health Sciences Centre, Research Ethics Board (study file number 023-2011).

## Results

There were 54 family physicians who participated in EMRALD as of 2008. A comparison of these 54 FPs to all FPs in Ontario, Canada is provided in Table 1. EMRALD FPs were located throughout Ontario. However, EMRALD FPs compared to all FPs in Ontario were younger, more likely to be female, a Canadian medical graduate and more likely to participate in a patient enrolment model. There was a higher proportion of EMRALD FPs from rural locations.

**Table 1:** Comparison of EMRALD family physicians with all other family physicians in Ontario, Canada

**Characteristic**	**EMRALD physicians**		**All other Ontario FPs**	
	**N**	**%**	**N**	**%**
Total	54	100.0	11,385	100.0
Sex				
Male	30	55.6	6,833	60.0
Female	24	44.4	4,552	40.0
Age group				
Under 35 years	9	16.8	1,094	9.6
35-54 years	33	61.1	5929	52.1
55+	12	22.2	4,362	38.3
Mean age (years)	44.9		50.6	
Medical training location				
Canada	47	86.6	8,731	76.7
US/International	7	13.4	2,654	23.3
Average number years in practice	14.0		17.0	
Rurality				
Rural	11	20.3	850	7.5
Suburban	8	14.8	1,871	16.4
Urban	35	64.8	8,664	76.1
More than 25% of visits in the Emergency Department*	10	18.5	1,560	13.7
Full time affiliation with a patient enrolment model group	54	100.0	6,866	60.3
Time on EMR				
<= 3years	18	33.3	NA	NA
3 to 5 years	26	48.2	NA	NA
> = 5 years	10	18.5	NA	NA
Rostered patients				
<=1000 patients	18	33.3	NA	NA
1001 to 1500 patients	20	37.0	NA	NA
> = 1501 patients	16	29.7	NA	NA

*FPs having more than 25% of the patient visits in an emergency department.

*NA* Not available.

### Wait times

The number of referrals in 2008 for each specialty type found in the EMR data and the proportion of these referrals successfully linked to an OHIP specialist full consultation claim are provided in Table 2. Over 80% specialty EMR referrals were associated with a specialist visit within the administrative data. However, only one third of mental health referrals within the EMR were associated with a psychiatrist OHIP visit/claim.

**Table 2:** Family medicine referrals from the EMR to administrative data specialist consultations visits

**EMR specialist category**	**Number of the referrals in EMR 2008**	**Number (percentage) of EMR referrals to the same or similar specialist with a consultation fee code found in the administrative data within two years**
Mental health	782	264 (33.76)
Rheumatology	422	351 (83.18)
Gastroenterology	2163	1876 (86.73)
Cardiology	754	644 (85.41)
Dermatology	2510	2162 (86.14)
Orthopedics	1129	913 (80.87)
ENT	1334	1086 (81.41)
Urology	888	680 (76.58)
Plastics	826	582 (70.46)
General surgery	1178	949 (80.56)

**Figure 1:**
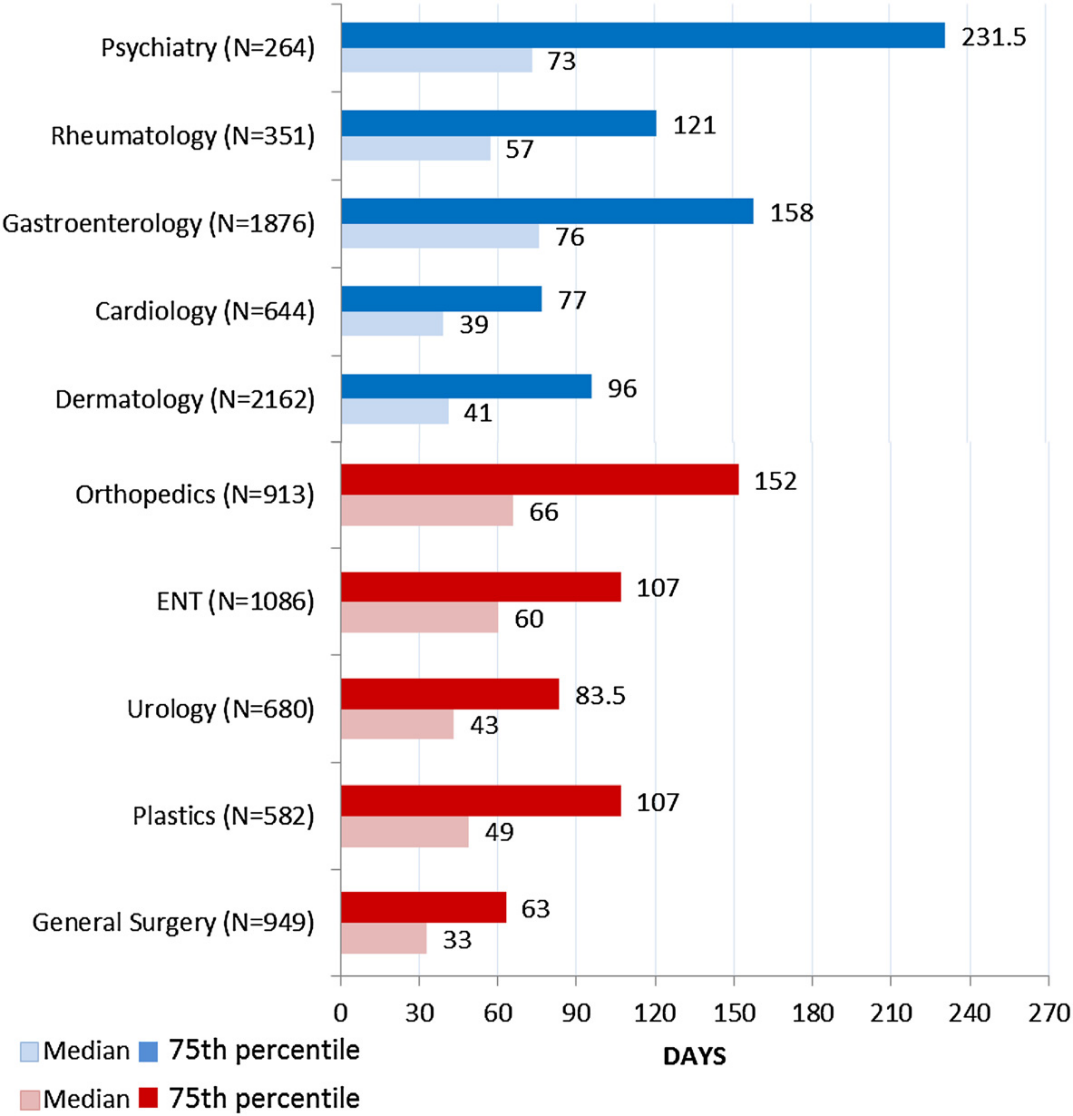
Wait times (in days) from a family physician referral to having a medical or surgical consultation visit.

The wait times, in days, from primary care to medical specialist and surgical specialist are presented in Figure 1. Cardiology had the shortest median medical wait time at 39 days and general surgery had the shortest median surgical wait time of 33 days. Gastroenterology had the longest median medical wait time at 76 days and orthopedics had the longest median surgical wait time at 66 days.

### Bivariate analyses

The bivariate analyses of wait times from a FP referral to seeing a specialist by patient factors are provided in Table 3. Patients with lower comorbidity had higher wait times to see a gastroenterologist. Female patients had higher wait times to see a rheumatologist, plastic or orthopedic surgeon, while male patients had a higher wait time for general surgery. Very old patients had shorter wait times than younger patients to see a gastroenterologist or ENT surgeon. Older patients had a longer wait time for rheumatology and orthopedic surgery. Patients in the highest income quintile compared to the lowest quintile had a longer wait time to see a gastroenterologist, but shorter wait times to see plastics or orthopedics. There were no statistically significant differences with wait times for comorbidity amongst on the surgical specialists or for continuity of care with a FP.

**Table 3:** Wait time (in days) from EMR referral to having a specialist visit: patient factors

**Patient factors**		**Dermatology**		**Gastroenterology**		**Rheumatology**		**Cardiology**		**Psychiatry**		**General surgery**		**Plastics**		**Urology**		**ENT**		**Orthopedics**	
		**Median**	**75th**	**Median**	**75th**	**Median**	**75th**	**Median**	**75th**	**Median**	**75th**	**Median**	**75th**	**Median**	**75th**	**Median**	**75th**	**median**	**75th**	**Median**	**75th**
**Sex**	**Female**	41	97	77	162	62*	127*	39	78	77.5	207	30*	60*	54*	117*	44.5	87.5	62	112	73.5*	159*
	**Male**	42	91	71	155	44.5*	90*	39.5	77	68	344	39*	71*	43*	100*	43	80.5	58.5	103	58*	141*
**Age groups (years)**	**<=20**	48	113	139*	187*	52*	147.5*	37.5	58	121	478	28	65	46	190	54.5	94	59*	101*	24*	39*
	**21 to 40**	40	92	71*	152*	59*	122*	25	64	62.5	231	35	69.5	49.5	123	46.5	81	53.5	98*	61*	157*
	**41 to 55**	42	92	79*	168*	57*	133*	42	76	71.5	204	33.5	67.5	50	108	48	87	61.5*	117*	62.5*	150*
	**56 to 65**	42	97	81*	174.5*	60*	116*	37	79	85	291	34	61	45	100	41	76	63*	116*	83*	173*
	**66 to 85**	37	96	60.5*	141*	56*	105*	44	78	123.5	322	29	55	55	99	41.5	88	65*	106*	71*	149*
	**> = 86**	50.5	99	25*	71*	33*	98*	27	121	35	56	41	68	29.5	40	33	43	48*	142*	37*	84*
**Socioeconomic status**	**1 (Low)**	38	89	70*	142*	57	122	41	77	101.5	385	33.5	64	73*	132*	39.5	79.5	54.5	98	99*	165*
	**2**	42	93	78*	155*	59	116	37	84	88	236	42	76	62*	140*	49	98	61	112	64*	144*
	**3**	48	98	63*	137*	55	116	35	62	75.5	220	25	59	49*	116*	41	77	58	110	62*	140*
	**4**	38.5	88	73*	152.5*	50.5	127	42	93	47.5	160	32	63	46*	99*	46	85	62.5	108	68*	152*
	**5 (High)**	41	109	84*	212*	69	124	42.5	78	48.5	128	30	60	41*	90*	43	81	60	111	54*	159*
**Comorbidity (ACG group)**	**0-5 (Low)**	42	93	81*	172*	59.5	119.5	37	68	60	243	33.5	62	49	108	45	77.5	64	113	65	150
	**6 to 9**	42	97	63.5*	146*	55.5	116	46	87	84	209	32	64	51	106	41.5	80	53	103	69	145
	**> = 10 (High)**	33	112	66.5*	138.5*	57	150	26	73	100	230	33.5	82	44	100	40	147	47	98	67	188
**Continuity of care (UPC)**	**Low UPC**	43	104	75	146	59	117	41.5	103	77.5	187	31	69	49	112	49	105	57	105	52	139
	**High UPC**	39	90	76	173	58	118	36.5	68	86	233	32.5	63.5	47	103	43	80	63	116	78	161
	**N0 UPC**	43	94	71	151	56.5	128.5	41	71	65	308	35	60	55	118	40	81.5	57	104	58.5	150

*p < 0.001.

The bivariate analyses of wait times from a FP referral to seeing a specialist by physician factors are demonstrated in Table 4. FPs in rural practices had longer wait times for referrals to urology and ENT. FPs in suburban practices had longer wait times for dermatology and orthopedic referrals. Metropolitan based FPs had longer gastroenterology and general surgery wait times. Male physicians had longer wait times for cardiology, dermatology and ENT referrals. Older FPs had longer wait times for gastroenterology referrals and shorter wait times for ENT referrals. Physicians practicing in capitation-based models had longer wait times for gastroenterology and general surgery referrals, but shorter wait times for rheumatology and orthopedic referrals. Wait times for referrals to dermatology and gastroenterology were longer as roster sizes for FPs increased.

**Table 4:** Wait time (in days) from EMR referral to having a specialist visit: physician factors

**Physician characteristics**		**Dermatology**		**Gastroenterology**		**Rheumatology**		**Cardiology**		**Psychiatry**		**General surgery**		**Plastics**		**Urology**		**ENT**		**Orthopedics**	
		**Median**	**75th**	**Median**	**75th**	**Median**	**75th**	**Median**	**75th**	**Median**	**75th**	**Median**	**75th**	**Median**	**75th**	**Median**	**75th**	**Median**	**75th**	**Median**	**75th**
**MD gender**	Male MD	55*	134*	70	149	58	120	43*	84*	65	188	33	60	53.5	108.5	42	77	67*	120*	70.5	155
	Female MD	35*	73*	80	175	57	121.5	35*	66*	87	326	33.5	70.5	45.5	106	47.5	94.5	53*	92*	60	141
**MD Age**	<=35 years	43	87.5	69*	144.5*	56	115	37	96	92	354	31	63	48	107.5	39.5	88	57	93	62	149
	36 to 55 years	41	103	76*	155*	62	125	41	76.5	70	233	36	72	43	104	45	81	67*	120.5	67	150
	> = 56 years	41	84	78*	178*	41.5	97	37	77	64	158	30	52	66.5	113.5	41	86	46*	98	64	157
**Practice location**	Metropolitan	39*	85*	81.5*	182*	52	116	42	83	80.5	232.5	36*	77*	48	112	39*	70*	60	106	55*	136*
	Suburban	73*	192*	40*	77*	69	133	28.5	84	117.5	344	24*	46.5*	55	102	34*	94.5*	55*	98*	89*	174.5*
	Rural	51*	168*	71*	127*	76	133	37	68	41.5	65	38*	68*	52	114	67.5*	140.5*	76*	142*	79*	174.5*
**Primary care model**	FHN/FHO	42	97	96.5*	205*	49*	115*	43.5	78	90.5	232.5	40*	82*	45	123	44.5	78	57	110	62*	141
	FHG/other	41	90	57*	121*	67.5*	130*	36	76.5	63	231.5	29*	53*	51	103	42	88	62	106	68.5*	163
**Rostered patients**	500 to 1000	37*	71*	66.5*	129*	56	147	46	78	60	239.5	37	78	49	100	41	80	49	99	62	137
	1001 to 1500	36*	73*	60*	136*	58	117	35	62	88	334	27.5	53	45	97	38.5	72	68	109.5	58.5	142
	> = 1500	50*	134*	90*	226*	55.5	119.5	43	86.5	72.5	187	37	68	55	125	48	95	59	110	67	150

*p < 0.001.

### Multivariate analyses

Multivariate analyses of patient factors for each medical and surgical specialty are provided in Table 5. Healthier patients had longer wait times for gastroenterology and ENT consultation visits and patients in lower income quintiles had shorter wait times for gastroenterology visits and longer wait times for plastics visits. Male patients had shorter wait times for plastics and rheumatology and longer wait times for general surgery. Higher continuity of care with a FP was associated with longer wait times for orthopedics. Older patients had longer waits for orthopedic consultations. No other patient factors were associated with medical or surgical specialist wait times.

**Table 5:** Multivariate analysis of patient factors and wait times

**Dermatology**	**Degree of freedom**	**Sum or square**	**Mean square**	**F statistic**	**p value**
Patient age	1	0.61	0.61	0.37	.054
Patient sex	1	0.17	0.17	0.11	0.74
Socioeconomic status	4	5.28	1.32	0.81	0.52
Patient ACG index	2	2.73	1.63	0.84	0.43
Continuity of care with their FP	2	2.08	1.04	0.64	0.53
Gastroenterology	Degree of freedom	Sum or square	Mean square	F statistic	p value
Patient age	1	5.96	5.96	4.08	0.044
Patient sex	1	1.33	1.33	0.91	0.34
Socioeconomic status	4	19.22	4.8	3.29	0.011
Patient ACG index	2	20.67	10.33	7.07	0.0009
Continuity of care with their FP	2	1.45	0.72	0.5	0.61
Rheumatology	Degree of freedom	Sum or square	Mean square	F statistic	p value
Patient age	1	0.025	0.025	0.02	0.89
Patient sex	1	8.86	8.86	7.44	0.0067
Socioeconomic status	4	5.38	1.34	1.13	0.34
Patient ACG index	2	0.44	0.22	0.19	0.83
Continuity of care with their FP	2	0.16	0.081	0.07	0.93
Cardiology	Degree of freedom	Sum or square	Mean square	F statistic	p value
Patient age	1	2.07	2.068	1.42	0.23
Patient sex	1	1.35	1.35	0.93	0.34
Socioeconomic status	4	3.02	0.75	0.52	0.73
Patient ACG index	2	4.18	2.08	1.43	0.24
Continuity of care with their FP	2	4.37	2.18	1.5	0.22
Psychiatry	Degree of freedom	Sum or square	Mean square	F statistic	p value
Patient age	1	0.21	0.21	0.1	0.75
Patient sex	1	0.9	0.9	0.45	0.51
Socioeconomic status	4	17.1	4.26	2.14	0.077
Patient ACG index	2	6.29	3.14	1.58	0.21
Continuity of care with their FP	2	2.29	1.14	0.57	0.56
General surgery	Degree of freedom	Sum or square	Mean square	F statistic	p value
Patient age	1	2.41	2.41	1.79	0.18
Patient sex	1	12.3	12.3	9.14	0.0026
Socioeconomic status	4	5.83	1.46	1.08	0.36
Patient ACG index	2	0.77	0.39	0.29	0.75
Continuity of care with their FP	2	1.73	0.86	0.64	0.53
Plastic	Degree of freedom	Sum or square	Mean square	F statistic	p value
Patient age	1	0.21	0.21	0.13	0.72
Patient sex	1	9.63	9.63	6.05	0.014
Socioeconomic status	4	17.8	4.46	2.81	0.025
Patient ACG index	2	2.55	1.28	0.8	0.45
Continuity of care with their FP	2	0.38	0.19	0.12	0.89
Urology	Degree of freedom	Sum or square	Mean square	F statistic	p value
Patient age	1	0.0064	0.0064	0.01	0.94
Patient sex	1	1.26	1.26	1	0.32
Socioeconomic status	4	1.1	0.28	0.22	0.93
Patient ACG index	2	1.98	0.99	0.79	0.45
Continuity of care with their FP	2	3.78	1.88	1.5	0.22
ENT (Otolaryngology)	Degree of freedom	Sum or square	Mean square	F statistic	p value
Patient age	1	0.42	0.42	0.32	0.57
Patient sex	1	5.74	5.74	4.36	0.037
Socioeconomic status	4	0.76	0.19	0.14	0.97
Patient ACG index	2	15.9	7.98	6.06	0.0024
Continuity of care with their FP	2	1.38	0.69	0.52	0.59
Orthopedics	Degree of freedom	Sum or square	Mean square	F statistic	p value
Patient age	1	21.2	21.25	12.3	0.0005
Patient sex	1	6.78	6.78	3.92	0.048
Socioeconomic status	4	15.9	3.99	2.31	0.56
Patient ACG index	2	2.11	1.06	0.61	0.54
Continuity of care with their FP	2	18.9	9.49	5.49	0.0043

Multivariate analyses of provider factors for each medical and surgical specialty are provided in Table 6. Rural FP practices had longer wait times for psychiatry and urology, while suburban FP practices had longer wait times for dermatology and orthopedics and urban FP practice had longer wait times for gastroenterology. FP practices having a higher number of patients were associated with longer wait times for dermatology, gastroenterology, urology and ENT. Male FPs and older FPs had longer wait times to dermatology and ENT. No physician factors associated with cardiology, rheumatology and plastics wait times.

**Table 6:** Multivariate analysis of physician factors and wait times

**Dermatology**	**Degree of freedom**	**Sum or square**	**Mean square**	**F statistic**	**p value**
Patient age	1	0.61	0.61	0.37	.054
Patient sex	1	0.17	0.17	0.11	0.74
Socioeconomic status	4	5.28	1.32	0.81	0.52
Patient ACG index	2	2.73	1.63	0.84	0.43
Continuity of care with their FP	2	2.08	1.04	0.64	0.53
Gastroenterology	Degree of freedom	Sum or square	Mean square	F statistic	p value
Patient age	1	5.96	5.96	4.08	0.044
Patient sex	1	1.33	1.33	0.91	0.34
Socioeconomic status	4	19.22	4.8	3.29	0.011
Patient ACG index	2	20.67	10.33	7.07	0.0009
Continuity of care with their FP	2	1.45	0.72	0.5	0.61
Rheumatology	Degree of freedom	Sum or square	Mean square	F statistic	p value
Patient age	1	0.025	0.025	0.02	0.89
Patient sex	1	8.86	8.86	7.44	0.0067
Socioeconomic status	4	5.38	1.34	1.13	0.34
Patient ACG index	2	0.44	0.22	0.19	0.83
Continuity of care with their FP	2	0.16	0.081	0.07	0.93
Cardiology	Degree of freedom	Sum or square	Mean square	F statistic	p value
Patient age	1	2.07	2.068	1.42	0.23
Patient sex	1	1.35	1.35	0.93	0.34
Socioeconomic status	4	3.02	0.75	0.52	0.73
Patient ACG index	2	4.18	2.08	1.43	0.24
Continuity of care with their FP	2	4.37	2.18	1.5	0.22
Psychiatry	Degree of freedom	Sum or square	Mean square	F statistic	p value
Patient age	1	0.21	0.21	0.1	0.75
Patient sex	1	0.9	0.9	0.45	0.51
Socioeconomic status	4	17.1	4.26	2.14	0.077
Patient ACG index	2	6.29	3.14	1.58	0.21
Continuity of care with their FP	2	2.29	1.14	0.57	0.56
General surgery	Degree of freedom	Sum or square	Mean square	F statistic	p value
Patient age	1	2.41	2.41	1.79	0.18
Patient sex	1	12.3	12.3	9.14	0.0026
Socioeconomic status	4	5.83	1.46	1.08	0.36
Patient ACG index	2	0.77	0.39	0.29	0.75
Continuity of care with their FP	2	1.73	0.86	0.64	0.53
Plastic	Degree of freedom	Sum or square	Mean square	F statistic	p value
Patient age	1	0.21	0.21	0.13	0.72
Patient sex	1	9.63	9.63	6.05	0.014
Socioeconomic status	4	17.8	4.46	2.81	0.025
Patient ACG index	2	2.55	1.28	0.8	0.45
Continuity of care with their FP	2	0.38	0.19	0.12	0.89
Urology	Degree of freedom	Sum or square	Mean square	F statistic	p value
Patient age	1	0.0064	0.0064	0.01	0.94
Patient sex	1	1.26	1.26	1	0.32
Socioeconomic status	4	1.1	0.28	0.22	0.93
Patient ACG index	2	1.98	0.99	0.79	0.45
Continuity of care with their FP	2	3.78	1.88	1.5	0.22
ENT (Otolaryngology)	Degree of freedom	Sum or square	Mean square	F statistic	p value
Patient age	1	0.42	0.42	0.32	0.57
Patient sex	1	5.74	5.74	4.36	0.037
Socioeconomic status	4	0.76	0.19	0.14	0.97
Patient ACG index	2	15.9	7.98	6.06	0.0024
Continuity of care with their FP	2	1.38	0.69	0.52	0.59
Orthopedics	Degree of freedom	Sum or square	Mean square	F statistic	p value
Patient age	1	21.2	21.25	12.3	0.0005
Patient sex	1	6.78	6.78	3.92	0.048
Socioeconomic status	4	15.9	3.99	2.31	0.56
Patient ACG index	2	2.11	1.06	0.61	0.54
Continuity of care with their FP	2	18.9	9.49	5.49	0.0043

## Discussion

We determined the median wait times from FP referral to seeing a specialist were from 5 to 11 weeks and the 75th percentile wait times from 9 to 33 weeks. With a few exceptions, patient factors were not consistently associated with wait times from primary care to having a specialist consultation visit. Similarly, FP practice size, FP sex and type of FP primary care enrolment model were not consistently associated with wait times. For many specialist physician types, FP practice location was associated with wait times from primary care.

Our wait times estimates are similar to published studies which are based on specialist physician self reports [31,54,55]. However, our measures of wait times for both medical and surgical specialists are greater than the 5 week and 2 week median wait times for non-urgent or urgent referrals previously reported for all specialist physicians together in Ontario in the National Physician Survey (NPS) report [32]. A comparison to NPS data is difficult because it includes all medical and surgical specialists together and we looked at specific specialist types. We also did not separate out urgent and non-urgent referrals. The NPS also rated accessibility to specialist types with a tendency for some medical specialists (psychiatry) to have a lower proportion of very good and excellent access than some surgical specialists (general surgery and ENT). The NPS estimates are based on physician opinions and may not in fact represent the reality of wait times from the patients’ perspective.

Our wait time measures are based on actual claims in the health care system. What differentiates EMRALD from other primary care EMR data sources in Canada is that it includes the entire FP EMR record and it is linked to the Ontario health administrative data. We were able to link over 80% of the EMR FP referrals to a physician claim. We limited our specialist visit claim to include a full consultation visit. Some of our FP EMR referrals may be for a reassessment visit and therefore were not linked to a full consultation claim. EMR mental health referrals had a low proportion linked to a psychiatrist claim. Many mental health referrals are made to non-physician providers such as psychologists or social workers who do not appear in the physicians claim data. For all patients, a certain proportion of referrals to specialist may be cancelled or result in a no show. This proportion may be higher for psychiatric referrals. Further work looking into the content of mental health referrals, including referrals for specific diagnoses, in currently underway.

We found that wait times from primary care to either medical or surgical specialist visits are not consistently related to patient factors. Other studies have found specialist visits and referrals rates to be associated with SES and comorbidity in Ontario [8,9]. Specialist physicians tend to triage referrals based on their urgency. However, urgency of the referral was not well documented and therefore not assessed in this study. The specific disease is likely to also dictate the urgency of a referral. For example, seeing a cardiologist for unstable angina would be more urgent than advice on better blood pressure management. Further determination of wait times for specific diseases or condition is needed.

Practice location is the most consistent influence on wait times. Busier practices may have higher referral rates and therefore longer wait times. As seen in other studies comparing patients seen in different primary care delivery models, differences were seen in wait times to specialists between capitation-based primary care models compared to other models [14]. Further work examining FPs participating in models which include health care providers may explain some of these differences. For example, FPs who work in practices which include physiotherapists or sport medicine therapists, may manage most of their musculoskeletal conditions thereby referring fewer patients and then have shorter wait times to orthopedics or rheumatology. While physician gender and age are associated with referrals rates in Ontario [8], they are not associated consistently related to wait times.

The Wait Time Alliance in Canada has recommended benchmarks for a select number of conditions or investigations [4]. For example non-urgent hip and knee replacement should be done within 10 months after seeing an orthopedic surgeon. However, it is important to get an understanding of the wait time from primary care. FPs need to be able to manage patients, with various levels of complexity, prior to having the opinion or diagnostic information from a specialist physician. Prolonged wait times to see a specialist physician will potentially change the burden of care for some patients from specialty care to primary care [56]. In Canada, more FPs belong to newer primary care delivery models, many of which include other health care providers [44]. If wait times become increasingly long, primary care delivery models will need to be structured to support the care of more complex patients. For example, if the wait to see an orthopedic surgeon or pain specialist is too long, then primary care practice may want to include physiotherapists who can address some aspects of patient care. If certain health care regions have longer wait times than deemed acceptable, local programs assisting in access to either outside regional care or access to other health care providers would be helpful with the regional planning of services.

Study limitations: We included a convenience sample of community based FPs, with a higher proportion practicing in rural locations. As FP practice location is related to wait times, further work which includes a larger sample of FPs needs to be undertaken. In our study we look at all types of referrals to specialists and we did not examine specific diseases or conditions. For example, it is likely the wait time to for more acute or unstable conditions would be faster than less serious conditions. We were not able to assess the priority or urgency of the referral. Finally the quality of the referral, including information contained in the letter and accompanying test results, was not assessed in this study.

## Conclusions

Wait times from primary care to specialty care are longer than those reported by physician surveys in Ontario, Canada with median waits from 33 to 76 days and 75th percentiles of 63 to 231.5 days. Wait times from primary to specialty care need to be included in the calculation of surgical and diagnostic wait time benchmarks in Canada. Patient factors and most physician factors do not seem to be consistently associated with wait times, except for FP practice location and practice size.

## Competing interests

The authors declare that they have no competing interests.

## Authors’ contribution

LJ prepared the first and final draft of the article. HL was primarily responsible for the data analysis. ES was a research associate for this paper and coded the EMR referrals into a specialty type. KT, JB, ES and RG revised the article critically for its content. All of the authors reviewed the article and approved the final version for publication.

## Pre-publication history

The pre-publication history for this paper can be accessed here:

http://www.biomedcentral.com/1471-2296/15/16/prepub
